# A Qualitative Template Analysis to Understand Patient and Practitioner Perspectives on a Psychological Intervention for Fatigue in Inflammatory Bowel Disease

**DOI:** 10.1002/jgh3.70363

**Published:** 2026-02-16

**Authors:** Catherine Emerson, Anna Klas, Peter R. Gibson, Lisa Olive, Matthew Fuller‐Tyszkiewicz, Antonina Mikocka‐Walus

**Affiliations:** ^1^ School of Psychology Deakin University Burwood Australia; ^2^ SEED Lifespan Strategic Research Centre Deakin University Burwood Australia; ^3^ Department of Gastroenterology, School of Translational Medicine Monash University and Alfred Health Melbourne Australia

**Keywords:** co‐design, colitis, Crohn's, fatigue, IBD, psychotherapy

## Abstract

**Aims:**

Fatigue in IBD is pervasive, with very few intervention options available to patients. Research has indicated that psychological therapies are a promising strategy for managing IBD fatigue. However, no such intervention is available to date. This study aimed to evaluate the opinion of patients and health professionals on a potential psychological intervention for IBD fatigue.

**Methods and Results:**

A total of seven patients (Crohn's disease = 5, ulcerative colitis = 2) and seven health professionals participated in semi‐structured interviews. Results were derived by using a template analysis, revealing four key themes and associated subthemes: (1) it's a trade‐off; (2) buy‐in, trust and credibility; (3) accountability; and (4) accessibility and equity. Important factors identified were a self‐led online intervention, where patients can converse with other patients. Patients determined a facilitator with IBD‐specific knowledge to be important. Health professionals were concerned with the cost‐effectiveness and duration of an intervention, whereas patients were less concerned about the financial cost if there is perceived benefit to the intervention.

**Conclusion:**

Findings indicate the need to balance support with accessibility for patients. The lack of consensus of who can deliver an intervention by health professionals is significant and underscores the need for mental health care to be integrated into IBD health services. Nurses were recommended as an alternative to mental health professionals. However, the nurses interviewed emphasized the impact this would pose on their workloads. As such, there needs to be a diversity of supporting staff in IBD health care teams to ensure that future psychological interventions are feasible.

## 1 Introduction

Of the many symptoms that confront patients with inflammatory bowel disease (IBD), fatigue, which is described as an overwhelming sense of tiredness and exhaustion that impacts patients' cognitive, mental and physical capacity [1], is common. Fatigue impacts the patient quality of life [2, 3] and may be even more debilitating and life‐limiting than their bowel symptoms [4]. Its prevalence is estimated to be 72% in six studies of patients with active disease and 47% when seven studies of clinical remission were pooled [5]. While fatigue is believed to be multifaceted resulting from active inflammation, micronutrient deficiencies, sleep disturbances, lifestyle factors (i.e., diet and smoking), and psychological comorbidity (e.g., depressive symptoms; [2, 6]), the effectiveness of therapeutic interventions for fatigue has been poor.

Management strategies should focus on identifying and addressing the individual contributing factors for each patient [6]. For example, when taking a biopsychosocial approach, for patients with a large proportion of biological factors driving their fatigue, inducing remission and addressing micronutrient deficiencies can help mitigate the experience of fatigue. However, as approximately 50% of patients in clinical remission still experience debilitating levels of fatigue [5], there is a need for interventions to also address the psychosocial contributors of fatigue (e.g., high levels of stress and depressive and anxiety symptoms).

Due to the often‐psychosocial underpinnings of IBD fatigue and the apparent benefits of psychological interventions for fatigue in patients with other chronic inflammatory conditions, like rheumatoid arthritis and multiple sclerosis [7, 8], such therapies should be an important management strategy. However, there has been limited development of psychological interventions to manage IBD fatigue [9]. Interventions such as cognitive behavior therapy (CBT), solution‐focused therapy, and psychoeducation have shown promising results for the improvement of IBD fatigue, but trials have been underpowered with short follow‐up periods, making it difficult to ascertain their true efficacy. Further, only one CBT intervention [10] included qualitative outcomes to evaluate patient perspectives, making it difficult to determine people's preferences for future interventions. While other chronic conditions can shed light on the possible benefits of psychological interventions like CBT, patients with IBD have their own symptomology and therefore require their own set of considerations when developing interventions. As such, there has been a growing interest in co‐developing interventions to meet the needs of population groups as this can potentially increase patient engagement and enhance intervention efficacy [11]. Involving key stakeholders, such as patients with IBD and health professionals, ensures interventions are tailored to the needs of the population and thus enhances intervention acceptability and engagement [12].

The aim of the current study is to explore the opinions of patients and health professionals on a potential psychological intervention for IBD fatigue. We employed a qualitative methodology as it allowed us to deeply explore and unpack their views concerning psychotherapy treatments for fatigue. Given the study was qualitative in nature, we did not generate a hypothesis. Instead, we developed the following research question to guide data analysis and interpretation: what are the perspectives of patients with IBD and IBD health professionals on psychotherapy treatments to target fatigue?

## 2 Methods

### 2.1 Participants

Patients were eligible to participate if: (1) they had a self‐reported diagnosis of IBD, (2) had a self‐reported history of fatigue, and (3) were over the age of 18 years. Health professionals were eligible to participate if: (1) they had worked in the field of IBD for a minimum of two years, and (2) were familiar with the symptom of fatigue. Details on recruited participants are outlined in Table 1.

**TABLE 1:** Demographic characteristics of participants.

	Pseudonym	Gender	Age	IBD patient?	Diagnosis	Education level	Occupation
1	Ellen	Female	46	Yes	Crohn's disease	Year 12	Client relationship manager
2	Tiffany	Female	32	Yes	Crohn's disease	Year 12	Waitress, receptionist and pyrotechnic
3	Jack	Male	28	Yes	Ulcerative colitis	High School	Commercial real estate agent
4	Ryan	Male	40	Yes	Crohn's disease	Master's degree	Financial services manager
5	Amelia	Female	21	Yes	Crohn's disease	Year 12	Student
6	Kate	Female	24	Yes	Ulcerative colitis	Honors degree	Occupational therapist
7	Ellie	Female	48	Yes	Crohn's disease	Bachelor's degree	Early childhood teacher
8	Chelsea	Female	40	No	—	Bachelor's degree	Gastroenterologist
9	Maggie	Female	60	No	—	Bachelor's degree	Nurse
10	Sarah	Female	34	No	—	Master's degree	Psychologist
11	Justine	Female	60	No	—	Master's degree	Psychologist
12	Nicole	Female	36	No	—	Post‐graduate certificate	IBD nurse
13	Sam	Male	48	No	—	Doctorate	Clinical psychologist
14	Ian	Male	70	No	—	N/A	Gastroenterologist

### 2.2 Study Design and Data Collection

The reporting of the study is guided by the SQRQ checklist [13], and is available as a checklist in the Supporting Information.

An epistemological position of critical realism was utilized, wherein focus was on describing participants' observable reality while acknowledging the influence of researchers' own perspectives of the observations described [14]. A descriptive theoretical framework was then used to understand the experiences of patients and health professionals. This meant that analysis stayed “close” to the surface meaning of the data, wherein participant responses were taken as they were, rather than interpretating beyond what participants said [15, 16]. See the Supporting Information for a reflexivity statement.

A two‐pronged approach to data collection was utilized. For patients, we sought out male and female IBD patients with a diagnosis of Crohn's disease and ulcerative colitis. The research team (CE) initially posted letters asking patients from a Melbourne (Australia) hospital IBD clinic to participate in an interview during December 2019 to January 2021. However, this method was curtailed by the start of the COVID‐19 pandemic, Melbourne lockdowns and social distancing measures. Convenience sampling via online IBD‐related social networks was then employed, with advertisements placed on the social media (Facebook, Instagram) accounts for Crohn's & Colitis Australia. For health professionals, we used a snowballing sampling technique where emails were sent by a senior member of the research team (AMW), targeting gastroenterologists, IBD nurses and psychologists. They were invited to participate or recommend a colleague who might be interested.

Potential participants who indicated interest were invited to participate in a telephone, face‐to‐face or online interview with CE. Of those who responded, *n* = 12 completed a telephone interview and *n* = 2 completed their interview online via Zoom, all using an audio recording to record the conversation. Informed consent was granted before interviews began, with a semi‐structured interview schedule used [17]. Questions were modeled off prior qualitative studies in the area [18, 19] and adapted for each participant group (see Supporting Information for questions). This involved showing participants an example of a potential intervention using materials from the “Tame Your Gut” CBT intervention that has previously been trialed in the IBD population [20]. The average interview length was 71 min, ranging between 60 to 90 min for patients; and an average of 54 min, ranging from 41 to 80 min for health professionals. While the interviewer initially endeavored to establish data saturation iteratively by noting the emergence of consistency in initial themes [21], this process was impacted by needing to stop data collection and initiate changes in recruitment in response to the Covid‐19 pandemic. As a result, the limited responses in recruitment forced the close of data collection. However, a systematic review determined we likely achieved data saturation as 9–17 interviews are deemed sufficient to reach saturation in studies with homogenous groups and narrow research objectives [22], as was the case in the current study.

All participants gave written informed consent and the protocol was approved by The Alfred Health HREC 455/19 and Deakin University DUHREC 2019‐363 in accordance with the principles of the Declaration of Helsinki. This study received no funding.

### 2.3 Data Analysis

All interviews were digitally recorded and transcribed verbatim, with identifying information changed during transcription for participant anonymity. Unnecessary details (e.g., repetitions) were also removed to increase data interpretability [14, 23]. Transcripts were then checked by CE for accuracy.

To identify patterns of meaning across the data, a thematic template analysis was performed to synthesize the interview data. A template analysis emphasizes the use of hierarchical coding, balancing structure in the analytical process with the flexibility to adapt coding as needed was performed [24]. It involved six steps as outlined by Brooks, McCluskey [25]: (1) CE became familiar with the data through re‐reading the transcripts, and AK read 25% of the data to provide a cross‐check of codes, (2) CE engaged in preliminary hand coding of the data, discussing these initially generated codes with AK, (3) CE and AK organized emerging themes from initial codes into meaningful clusters, (4) CE developed the coding template, (5) CE applied the template to 10% of the data and modified it as necessary, and (6) CE finalized the template with AK and applied it to the full data set, with any discrepancies resolved through discussion. Once the data was fully coded by CE, a final written summary of themes was produced and provided to AK, PG, and AM‐W. for peer review. Upon feedback from these authors, a final reconfiguration of the themes occurred, where subthemes were drawn out of the core themes to help better represent the complexity of the interview data. These multiple reviews and feedback rounds, where codes and themes were further refined, can be considered a form of “peer debriefing” and were done to help increase the trustworthiness of the qualitative analysis [26]. All participants were invited to member check their responses and declined to do so.

## 3 Results

### 3.1 Participants

Patients with IBD (*n* = 7) and IBD health professionals (*n* = 7) were recruited, with a mean age of 42 (range 21 to 70) years and 10 being female. Further demographic details are presented in Table 1.

### 3.2 Synthesized Themes

Four synthesized themes were identified across patients and health professionals. Further sub‐themes were identified demonstrating distinct important aspects of intervention development. A summary is presented in Tables 2, 3, 4, 5 where we provide a short definition and exemplar quote for each theme and subtheme. Please see the Supporting Information for a detailed table of quotes.

**TABLE 2:** Theme 1 definition, subthemes and illustrative quotes.

Theme	Definition	Subtheme	Quote
Theme 1: It's a balancing act	Provides an outline of how patients and health professionals alike noted that, when developing a psychological intervention, there is a need to weigh up what is ideal and what is practical/feasible for the program, the patient and the resources available.		“A multifaceted approach is ideal. But I guess it depends on at what point does the patient become a bit saturated? You need to get them on side and say we want you to do these different elements, but you want it to be incorporated into their everyday life where they're not being completely saturated” (Chelsea, Gastroenterologist)
		Intervention time	“I would do it indefinitely … like I'm happy to do stuff that I know will give a good outcome to either myself or other people “(Ellen, Crohn's disease)
		Balanced cost	“If it's something that would benefit me and I know that it's something that will better wellbeing then yeah I would pay but … again it would just depend on the cost and what I would get for it” (Ellen, Crohn's disease) “Well then you're dealing with a cohort who have a lot of associated costs in terms of medication and other things … for instance where we have patients that we refer to the GP to get a mental health plan … they often don't go to the GP, and if they do go to the GP and get a plan they often don't enact it … and if they do enact it and there's out of pocket costs they don't keep it going.” (Maggie, IBD nurse)
		Who has responsibility	“I think probably like one of the reasons I haven't had much success in therapy is because pretty much all the things I'm going to therapy for are to do with my Crohn's, and I would spend the hour explaining Crohn's to … they wouldn't have much time to give me skills to cope with it.” (Amelia, Crohn's disease) “All those things are less of a priority on my list so sometimes I don't get to them because it's not much of a priority” (Chelsea, gastroenterologist)

**TABLE 3:** Theme 2 definition, subthemes and illustrative quotes.

Theme	Definition	Subtheme	Quote
Theme 2: Buy‐in, credibility and trust	Demonstrates how both patients and health professionals explained that the program must appear credible and trustworthy. It was emphasized this was especially needed when encouraging patients to sign on to the intervention as this perceived credibility would increase the likelihood that they were willing to try it, and trust that they would get something meaningful and useful from the program.		“Having a source of truth as well from a credible source cos there's so much RUBBISH out on the internet that you could read but its unqualified opinion.” (Ryan, Crohn's disease)
		Visual appearance	“I like to read but‐ I mean I know that especially these days people see a whole lot of sentences and paragraphs and if there's too much visually it just seems like too hard.” (Jack, Ulcerative colitis)
		The need for a deep understanding of IBD	“I'd like it to be someone who can relate to IBD so someone who has either studied it for a long period of time or someone like yourself who is doing this research” (Ellen, Crohn's disease)
		Rapport building	“You're not just a number … like if you have to walk into the room and they've forgotten everything about you and they have to read your file type thing … it kind of feels like they don't care” (Tiffany, Crohn's disease)
		Evidence‐based	“If you got something credible and that … you're saying this is from academia or something else it could be peer reviewed or have some basis in research… it would be nice to have a credible source” (Ryan, Crohn's disease)

**TABLE 4:** Theme 3 definition, subthemes and illustrative quotes.

Theme	Definition	Subtheme	Quote
Theme 3: Accountability	Represents how participants identified that engaging in a psychological program, while beneficial, would be difficult to adhere to. Both patients and health professionals alike suggested that juggling the intervention with other commitments (e.g., work, parenting requirements) would be challenging and therefore patients would require several strategies to help them remain engaged and accountable.		“Because then I'm made accountable … if you wrote up a plan for me then you've gone through the effort to do that plan and if I don't do it then I'm accountable and telling you now I will do it if it's incorporated into something … but if you say oh you know what just do it once a week then that won't motivate me … I think it would motivate me if I was held accountable.” (Ellen, Crohn's disease)
		Social interaction	“Yeah, and I think just especially if it was like I don't know you know an in‐person thing … say every month or couple of months, then I'd probably be more motivated to keep it up cause I would want to go to that.” (Kate, Ulcerative colitis)
		Adherence difficulties	“if you had to engage with somebody I don't think it would be overwhelming if you knew that things were going to be spaced out that you knew when it's coming up … like I had to do a module … and I know I had to have them done by the 17th of march … I know the expectations that I had to do them by that date … and I know I better get cracking on the reading … so sometimes having a due by date is good.” (Ellie, Crohn's disease) “These patients are already seeing dieticians, rheumatologists, infectious diseases, dermatologists, their gastroenterology, their surgeon, their GP. So, you know, another appointment out of their lives is very difficult.” (Maggie, IBD nurse)

**TABLE 5:** Theme 4 definition, subthemes and illustrative quotes.

Theme	Definition	Subtheme	Quote
Theme 4: Accessibility and Equity	Encapsulates patient and health professionals shared perspective that the program must be as accessible, equitable, and flexible as possible to ensure that patients can adhere to the program more easily and complete it successfully. For example, participants suggested that the program could be accessible via computer and smartphone, having telephone check ins with the program facilitator and keeping any activities short.		“I reckon that phone calls would be good for me personally … just cause I'm a bit busy and stuff like that … then I can do it if I'm at home or whatever … if I've had a busy day and then I don't want to go back out again and meet somebody … I can do a phone conversation … I can do it from home.” (Tiffany, Crohn's disease)
		Benefits and limits of self‐led interventions	“it just depends because I live in a remote part of Victoria … so where I would have to travel to and when I would have to travel and for how long so I'm so busy with work at the moment that if I was to do any of this it would be after hours … and my after hours are so precious to me at the moment now that I just want it to be about me.” (Ellen, Crohn's disease)
		Digital literacy	“I think if you gave this idea to people some might go I'm actually illiterate so they'll just go away because they'll go well that's no good to me … and they miss out on people to help them because there are barriers … and we have to cater for that person.” (Nicole, IBD nurse)
		Financial cost	“That's one of the number one things that you hear from them … they struggle with the cost and usually they're going to so many appointments that there might be other health conditions that they're managing as well … and the cost is a significant deterrent to any program that was going to cost them extra money.” (Sarah, Health psychologist)

### 3.3 Theme 1: It's a Balancing Act

A prominent theme was the concept that weighing up competing demands is essential when developing and implementing a feasible and useful psychological intervention for IBD‐related fatigue. Three subthemes were observed, with quotes presented in Table 2.

#### 3.3.1 Intervention Time

Health professionals highlighted the need to have short intervention times to reduce participant dropout. However, patients seemed to perceive intervention length differently. Some patients were hesitant to engage in an intervention that they perceived was long, but were willing to accept a longer duration if it increased intervention efficacy. Others were willing to complete the intervention for any length of time so long as they perceived they would gain improvements in their experience of fatigue. Additionally, some patients and health professionals shared the notion that the intervention needed to run for sufficient time to be effective.

#### 3.3.2 Balanced Cost

Most participants referred to the need to balance costs, including cost of time and finances. Health professionals emphasized the issues surrounding cost, either stating the need to make it worth patient time, subsidize the program, or generate service fees to fund the program. Nurses demonstrated the most awareness that an additional cost may be a barrier for patient engagement. Patients disclosed, however, that they were willing to pay if they gained something meaningful from the program.

#### 3.3.3 Who Has Responsibility

All health professionals named a psychologist as being the most ideal, yet it was also acknowledged that there are few gastro‐psychologists available in Australia. It was also unanimously acknowledged that a gastroenterologist could not take on this role due to time constraints and limited mental health experience. Other suggestions included social workers and general practitioners. However, IBD nurses were the most recommended despite nurses feeling like they were not qualified or their workload was already at capacity. In contrast, patients strongly emphasized that they wanted the person who drove the intervention to simply know and care about their illness and/or understand fatigue. Some patients even implied that they had negative prior experiences with health professionals and psychologists who did not know IBD.

### 3.4 Theme 2: Buy‐In, Credibility, and Trust

Patients and health professionals alike expressed the need for the program to appear credible and trustworthy for them to be willing to recommend or use the intervention. Four subthemes were identified, with quotes presented in Table 3.

#### 3.4.1 Visual Appearance

For patients, the program needed to look inviting and up‐to‐date with current visual standards. Patients also emphasized the importance of their fatigue being at the forefront of the materials, so that it was clear that their needs and limited mental and/or physical capacities were considered when creating the program. This contrasted with health professionals, who thought the example materials as presented were appropriate, although they also believed that careful consideration should be made for a visually appealing program. It was also highlighted that ultimately the intervention should align with the needs of patients by including their perspectives in the development to increase adherence and engagement.

#### 3.4.2 The Need for a Deep Understanding of IBD

For patients, having a facilitator who had a deep understanding of IBD or fatigue running any therapeutic sessions as a part of an intervention was important to enhance their trust and their likelihood to engage, as also noted above.

#### 3.4.3 Rapport Building

Health professionals echoed the importance of rapport building for developing patient trust in an intervention. Specifically, in the discussion of creating a blended intervention with online and facilitated components, psychologists expressed concern toward solely online interventions and whether they can communicate care. This emphasized that building a connection with the patient is essential for patient buy‐in and completion.

#### 3.4.4 Evidence‐Based

It was important to patients and health professionals alike that the program appeared evidence‐based and reputable. Health professionals emphasized the need for reputable endorsements, while patients outlined that including data to demonstrate the benefit of the program was something that would encourage buying into the program. For patients, this was seen in relation to justifying having to pay a financial cost, since living with IBD already had a considerable financial burden from their condition. Additionally, patients highlighted that data would enhance their understanding of the purpose of each exercise, increasing their willingness to adhere to the intervention.

### 3.5 Theme 3: Accountability

The idea of accountability on the part of the patient was often highlighted by both patients and health professionals, such that the intervention would keep patients engaged and adhering to the intervention protocol. Two subthemes were identified, with quotes presented in Table 4.

#### 3.5.1 Social Interaction

Social interaction was seen as a solution to enhance intervention adherence by patients and health professionals. Group therapy sessions were perceived by some as motivating as it would allow them to connect with other similar patients. The benefit of encouraging social interaction was also acknowledged by some health professionals. In fact, a common suggestion to help increase the physical activity of patients was to outline it as an opportunity for socializing, or to include group therapy to connect with other patients.

#### 3.5.2 Adherence Difficulties

Health professionals and patients alike suggested it would be challenging to participate in a program while managing their fatigue and busy lives. Health professionals also described the need for an intervention to imbed strategies to keep patients with IBD adherent to each component, as some had prior experiences with their patients not completing medical tasks set for their disease management. Health professionals outlined that including strategies to keep patients motivated and accountable despite their disease management plans would be a unique challenge to developing a psychological intervention in this population. Some health professionals suggested including objective ways of measuring adherence, such as the use of activity trackers to monitor patient activity levels or engagement with the intervention or using a due date as a viable solution.

### 3.6 Theme 4: Accessibility and Equity

Participants noted that the intervention needed to be as accessible and flexible as possible to enhance intervention success. Three subthemes were identified, with quotes presented in Table 5.

#### 3.6.1 Benefits and Limits of Self‐Led Online Interventions

The patient group emphasized the benefits of having a primarily self‐led online intervention supplemented with telephone or face‐to‐face therapeutic sessions as it would permit a certain degree of self‐determination and convenience. However, it was widely recognized by patients and health professionals that a solely self‐led online intervention may not be effective. There was a lack of consensus regarding how often patients should discuss content with a facilitator and in what format. Health professionals emphasized the benefit of using telehealth appointments as a means of facilitating the intervention. Being able to ask questions during a self‐led intervention was another important aspect of accessibility, with health professionals expressing concern that patients need to seek clarification or they may stop engaging. Patients echoed the need to seek further clarification while completing self‐led aspects, as some expressed that they may not feel confident in understanding the materials or that they may get stuck in trying to progress.

#### 3.6.2 Digital Literacy

Health professionals highlighted equity issues with the development of a blended fatigue intervention as not all patients are digitally literate or have access to a computer or the internet. Some patients mirrored this concern and suggested including a print‐function for online materials may ensure that the intervention facilitators can print and provide resources to those with low digital literacy.

#### 3.6.3 Financial Cost

As outlined in theme 1, the cost of the intervention was emphasized by health professionals as being a potential issue regarding accessibility and equity. To be fully equitable, the program needed to be free or heavily subsidized to allow access to all patients with IBD.

## 4 Discussion

The lack of strategies to ameliorate fatigue in patients with IBD is a major gap in the therapeutic armamentarium that has otherwise expanded. From our current understanding of the multifactorial nature of fatigue, a psychological approach will be the most appropriate and likely to succeed. However, to design such an approach requires engagement from health professionals and patients, taking into account the challenges associated with such therapeutic strategies. A first step is to define the multiple aspects that need to be addressed in the construction of any such program. In the current study, we employed qualitative methodology that permits a deep exploration of many aspects of the views of both patients and health professionals concerning psychotherapy treatments for fatigue in the Australian healthcare context. Four clear themes were identified and comprised balancing the ideal with the practical, gaining credibility and buy‐in, incorporating accountability to further enhance engagement, and defining issues of access and equity.

From a pragmatic point of view, balance has to be achieved between the design and delivery of a psychological program and the practical challenges it presents. The challenge of finding the right balance is reflected in a meta‐regression where a statistically significant association between number of psychotherapy sessions and efficacy was observed, but where the incremental effect size each additional session is very small [27]. Dealing with the costs of the intervention is a challenge given the general lack of such resources available to patients [2]. Nurses were particularly cognisant of this issue, potentially due to their close role in working alongside patients and advocating for their needs [28]; particularly given the cost of medication and medical care, and the difficulty in gaining government funding for programs. However, from the patient's point of view, the financial cost of an intervention is less of a concern as long as the invested financial cost would benefit their fatigue. However, it must be noted that this may be constrained by the Australian context that allows for publicly funded IBD care, with the majority of patients with IBD experiencing large healthcare costs globally [29]. Additionally, patients with IBD highlighted that psychological therapy is ideally delivered by a psychologist with a deep understanding of the underling disease, which is not typical in IBD care [30]. Previous work had reported that patients with IBD preferred engaging in services from health professionals who understood their disease‐specific needs and experiences (e.g., GPs, gastroenterologists) than those who lacked disease‐specific knowledge (e.g., dietitians, psychologists) [31]. Hence, a compromise between knowledge and understanding of the illness, and time and skills to deliver the therapy seemed supported by health professionals and patients alike.

There is a profusion of commercial programs on the internet that purport to provide solutions to many problems experienced by patients with IBD. It was not surprising then that patients and health professionals alike pointed to the need for any psychological program for fatigue to appear not only visually inviting, but also reputable, engaging, and with evidence of effectiveness. A difference in opinion between patients and health professionals about the appearance of the program pointed to the need for patient engagement in the program design to ensure that the “insider” perspective on the experience of having fatigue was included. Such inclusion would ultimately enhance the trust of patients and health professionals alike in the program [32, 33]. A recurring complaint of patients was their experience of having to explain their illness to progress with the intervention. As such, the importance for the experiences of those with IBD to be at the center of any care they receive as a part of a program was imperative for patient engagement and upkeep. Regarding the issue of blending online and facilitated components in the program, psychologists expressed concern toward solely online interventions and whether they can communicate care. This emphasized that building a connection with the patient is essential for patient buy‐in and completion. However, while building rapport is important [34], building therapeutic relationships is also possible in telehealth and online psychotherapy [35]. The final and key component of achieving buy‐in by patients and health professionals was the inclusion of reputable evidence of effectiveness of any program, which was particularly important if the program had financial cost to the patient. However, there was no agreement about the nature and quality of this evidence that was needed to reassure patients.

In order to enhance ongoing engagement and adherence, building accountability strategies into the program was suggested. This might be centered around social support via group sessions and/or group physical activity, or simply encouragement to block out time from their other responsibilities. This aligns with research that suggests the benefits of group‐based psychotherapy may include positive peer‐modeling opportunities, reinforcement of skills and social support [36]. A recent qualitative study highlighted that busy lives on top of their illness would impact people's with IBD motivation to complete an acceptance and commitment therapy intervention for associated distress [19]. However, similar to the present study, the sample of people with IBD was predominantly female and did not capture the male perspective. As the division of labor in a household is typically skewed toward women [37], it is difficult to determine how gender‐based dynamics influence the time people with IBD can invest in an intervention. As such, this demonstrates the desire for female patients with IBD to improve their fatigue while showing awareness that trying to sustain an additional activity on top of their current daily responsibilities will require strategies to keep them accountable.

Any program to ameliorate fatigue needs to be accessible and equitable. While patients emphasized the importance of self‐empowerment by having a major online component to the program, it was agreed by all that this had to be combined with other resources. As fatigue is known to reduce the cognitive capacity of patients with IBD [1], being able to refer to materials would aid in patients' consolidation of resources. Engagement with facilitators or other health professionals was considered important, but face‐to‐face interaction may create major issues in accessibility. Problems in equity include the cost that might be associated with the program and adaptation for lack of digital literacy, or access to a computer or the internet. This was a concern highlighted in a similar study by Dober, Mikocka‐Walus [19], where older adults with IBD may find it challenging to adhere to an online intervention due to digital literacy issues. As IBD tends to have a second wave of onset in patients aged between 60 and 70 years old [38], this could mean that a proportion of IBD patients would be unable to engage in an online intervention for their fatigue.

A limitation of the present study was the pandemic‐related recruitment constraints, limiting our ability to obtain a clinical sample, and doctors and nurses who work in medical settings, limiting the ability to establish data saturation through iteratively conducting interviews and identifying emerging themes. The lack of male participants further constrained the richness of perspectives. As it is known that males are difficult to engage in psychological services [39], a reduction in male perspectives makes it difficult to know how to enhance male patient engagement and accommodate their needs in a psychological intervention for IBD fatigue. Additionally, the perspectives collected can only represent those who work and experience healthcare within an Australian context, and therefore cannot adequately represent accessibility needs of people with IBD globally. Finally, the interviews would have been enhanced by asking patients if they had previous experience with psychotherapy. Therefore, it is difficult to determine how participant responses were shaped by any previous psychotherapy experiences.

A major implication of the present study was the strong emphasis of patient‐centered care. As such, for any intervention to be successful in retaining patients, careful consideration into their experience with fatigue needs to be implemented during the design process to ensure the intervention does not exacerbate fatigue. This is in line with recent research, which advises taking a patient‐centered approach by including early engagement with patients to improve the acceptability of trial designs [40]. In congruence with a fatigue‐centered approach, offering accessible ways of engaging with facilitators such as telehealth approaches and self‐led app or web‐based may reduce the added participatory burdens of travel, attending appointments, and impeding on personal responsibilities by offering flexibility for engagement. Further, the lack of psychologists trained in gastroenterology poses a significant issue for the success of a fatigue intervention. As patients expressed the importance for facilitators to know and understand IBD, strategies such as specialized training modules are needed to ensure that whoever facilitates the intervention is up to date on key disease‐specific knowledge. While patients with IBD did not specify any specific duration or frequency of sessions, it was emphasized that the benefit of each module needed to be apparent to keep patients engaged in the intervention. Additionally, accountability mechanisms like peer check‐ins and deadlines for completion of modules may buffer against lack of adherence and drop‐out.

In conclusion, this study offered insights into patient and health professional perspectives on a potential psychological intervention to manage IBD fatigue. The findings outlined the importance of an intervention to be designed around the reduced cognitive capacities IBD patients experience due to fatigue. The need for multi‐disciplinary care teams was emphasized. While duration of an intervention was flagged as a potential barrier for engagement by health professionals, patients were less concerned about duration if they could see they were gaining something meaningful. Finally, health professionals emphasized the need for an intervention to be cost effective, while patients were more willing to pay an out‐of‐pocket cost if they believed the financial trade‐off was worth it.

## Conflicts of Interest

A.M.‐W. has served as an educational speaker for Janssen and Ferring. C.E. has served as a patient advocate for Janssen. P.R.G. has served as a speaker, a consultant, and an advisory board member for Allergan, Janssen, MSD, Pfizer, Anatara, Atmo Biosciences, Immunic Therapeutics, Novozymes, and Takeda and Bristol‐Meyers Squibb, and has received research funding from MSD and Atmo Biosciences; and P.R.G. owns stocks and shares in Atmo Biosciences. The remaining authors declare no conflicts of interest.

## Supporting information


**Data S1:** Supporting Information.

**Data S2:** Supporting Information.

## Data Availability

Research data are not shared.
