# Supplementary material for: A Qualitative Template Analysis to Understand Patient and Practitioner Perspectives on a Psychological Intervention for Fatigue in Inflammatory Bowel Disease
Source: JGH Open. 2026 Feb 16;10(2):e70363. doi: 10.1002/jgh3.70363 (PMC12907766; doi:10.1002/jgh3.70363)
Supplement: Supplementary file 1 — Data S1: Supporting Information. [file JGH3-10-e70363-s001.docx]

# Supplementary Materials

## Reflexivity Statement

Regarding reflexivity, whereby researchers actively acknowledge how their position within the project may influence data interpretation, all authors resided in Australia and were experiencing the same COVID restrictions as participants and fall within the same healthcare system. However, the research included insider and outsider perspectives toward IBD fatigue. The primary researcher (CE) drew on her own experiences living with IBD to guide the interview process with patients and health professionals, which would have actively contributed to the interpretation of the data. AMW and PRG additionally have experience with IBD in a professional sense, and a personal sense (AMW), which contributed to the development of the project. Contrastingly, AK, LO and MFT served as outsider perspectives to the field and experience of IBD. The research team also contained expertise across a variety of disciplines and research areas, including health psychology (CE, AMW, LO), clinical psychology (LO), medicine (PRG), social psychology and qualitative research (AK) and online intervention and co-development intervention research (MFT), thus all shaping and contributing to the interpretation of the data in their own professional processes and expertise.

| **Supplementary Table**  *Interview schedule for patients and health professionals* | |
| --- | --- |
| Questions for Patients | Questions for Health Professionals |
| *Introduce ‘Tame Your Gut’ website and cognitive behavioural therapy*  How do you feel about using a website as an intervention to manage your fatigue? | *Introduce ‘Tame Your Gut’ website and cognitive behavioural therapy*  What are your thoughts on using a website for a fatigue intervention? Do you think it’s easy to use? |
| What are your thoughts on the website visually? | What are some potential barriers that you can think of that might stop patients from using a website? |
| Can you think of anything/any barriers that would stop you from using a website for a fatigue intervention? | *show example CBT activities*  What do you think of these activities? Do you think they’re user friendly and easy to understand? |
| *Show example CBT activities*  What do you think about these activities that could be a part of an intervention program? | What are some things you like about these activities? What are some things you dislike about the activities? |
| Are there any barriers that would prevent you from doing activities like these as a part of a program? | Can you see patients using these activities and why/why not? |
| Is there anything you can think of that would improve these activities? | Who do you think should deliver these activities? |
| How do you feel about doing physical activity as a part of a fatigue program? | What are your thoughts about incorporating physical activity in a fatigue intervention? |
| What are some barriers that could prevent you from engaging in physical activity as a part of an online program? | What are some barriers that may prevent patients from doing physical activity as a part of an intervention? |
| Would you prefer to do the program mainly online or face-to-face? | How many weeks do you think the intervention should run for? Why? |
| What do you think the balance should be between face-to-face and online sessions? Why do you think this? | How many sessions/activities do you think is feasible for patients to do/attend per week? Why? |
| Who do you think should deliver the program/any activities? Why? | How many sessions do you think should be in-person versus online per week? Why? |
| How many weeks do you think the intervention should run for? Why? | What are some barriers you can see patients having that could impact their ability to participate in the intervention? |
| How much time could you spend per week on an intervention? | What are your thoughts on cost? How much should the program cost? |
| What barriers can you think of that would prevent you from completing the intervention? |  |
| Would you be willing to pay for an intervention? How much do you think would be feasible? |  |
|  |  |

| **Supplementary Table**  *SRQR Checklist* | | | |
| --- | --- | --- | --- |
| **No.** | **Topic** | **Item** | **Page Number** |
|  | **Title and Abstract** |  |  |
| S1 | Title | Concise description of the nature and topic of the study Identifying the study as qualitative or indicating the approach (e.g., ethnography, grounded theory) or data collection methods (e.g., interview, focus group) is recommended | Page 1 |
| S2 | Abstract | Summary of key elements of the study using the abstract format of the intended publication; typically includes background, purpose, methods, results, and conclusions | Page 3 |
|  | **Introduction** |  |  |
| S3 | Problem Formulation | Description and significance of the problem/phenomenon studied; review of relevant theory and empirical work; problem statement | Pages 4-5 |
| S4 | Purpose of Research Question | Purpose of the study and specific objectives or questions | Page 5 |
|  | **Methods** |  |  |
| S5 | Qualitative Approach and Research Paradigm | Qualitative approach (e.g., ethnography, grounded theory, case study, phenomenology, narrative research) and guiding theory if appropriate; identifying the research paradigm (e.g., postpositivist, constructivist/ interpretivist) is also recommended; rationale** | Page 6-7 |
| S6 | Researcher characteristics and reflexivity | Researchers’ characteristics that may influence the research, including personal attributes, qualifications/experience, relationship with participants, assumptions, and/or presuppositions; potential or actual interaction between researchers’ characteristics and the research questions, approach, methods, results, and/or transferability | Supplementary materials |
| S7 | Context | Setting/site and salient contextual factors; rationale | Page 7 |
| S8 | Sampling Strategy | How and why research participants, documents, or events were selected; criteria for deciding when no further sampling was necessary (e.g., sampling saturation); rationale** | Pages 6-7 |
| S9 | Ethical issues pertaining to human subjects | Documentation of approval by an appropriate ethics review board and participant consent, or explanation for lack thereof; other confidentiality and data security issues | Page 8 |
| S10 | Data collection methods | Types of data collected; details of data collection procedures including (as appropriate) start and stop dates of data collection and analysis, iterative process, triangulation of sources/methods, and modification of procedures in response to evolving study findings; rationale** | Pages 6-7 |
| S11 | Data collection instruments and technologies | Description of instruments (e.g., interview guides, questionnaires) and devices (e.g., audio recorders) used for data collection; if/how the instrument(s) changed over the course of the study | Page 7 |
| S12 | Units of study | Number and relevant characteristics of participants, documents, or events included in the study; level of participation (could be reported in results) | Page 6; page 9 |
| S13 | Data processing | Methods for processing data prior to and during analysis, including transcription, data entry, data management and security, verification of data integrity, data coding, and anonymization/de-identification of excerpts | Pages 8-9 |
| S14 | Data analysis | Process by which inferences, themes, etc., were identified and developed, including the researchers involved in data analysis; usually references a specific paradigm or approach; rationale** | Pages 8-9 |
| S15 | Techniques to enhance trustworthiness | Techniques to enhance trustworthiness and credibility of data analysis (e.g., member checking, audit trail, triangulation); rationale** | Pages 8-9 |
|  | **Results/findings** |  |  |
| S16 | Synthesis and interpretation | Main findings (e.g., interpretations, inferences, and themes); might include development of a theory or model, or integration with prior research or theory | Pages 9-14 |
| S17 | Links to empirical data | Evidence (e.g., quotes, field notes, text excerpts, photographs) to substantiate analytic findings | Pages 9 - 14 |
|  | **Discussion** |  |  |
| S18 | Integration with prior work, implications, transferability, and contribution(s) to the field | Short summary of main findings; explanation of how findings and conclusions connect to, support, elaborate on, or challenge conclusions of earlier scholarship; discussion of scope of application/generalizability; identification of unique contribution(s) to scholarship in a discipline or field | Pages 14-18 |
| S19 | Limitations | Trustworthiness and limitations of findings | Page 18 |
|  | **Other** |  |  |
| S20 | Conflicts of interest | Potential sources of influence or perceived influence on study conduct and conclusions; how these were managed | Page 1 |
| S21 | Funding | Sources of funding and other support; role of funders in data collection, interpretation, and reporting | Page 8 |

| **Supplementary Table**  *Themes, sub-themes and illustrative quotes* | | | |
| --- | --- | --- | --- |
| **Theme** | **Definition** | **Subtheme** | **Quote** |
| Theme 1: It’s a balancing act | Provides an outline of how patients and health professionals alike noted that, when developing a psychological intervention, there is a need to weigh up what is ideal and what is practical/feasible for the program, the patient and the resources available. |  | “A multifaceted approach is ideal. But I guess it depends on at what point does the patient become a bit saturated? You need to get them on side and say we want you to do these different elements, but you want it to be incorporated into their everyday life where they’re not being completely saturated” (Chelsea, Gastroenterologist) |
|  |  | Intervention time | “I think that the intervention needs to be as short as it possibly can and not drag out because then the commitment becomes too big and then people don’t commit at all and then they might drop out halfway through … as far as weeks go maybe four to six.” (Nicole, IBD nurse)  “I would do it indefinitely … I would do it until you say I’m sick of you I don’t want to do this like I’m happy to do stuff that I know will give a good outcome to either myself or other people “(Ellen, Crohn’s disease)  “That’s a good question I think- talking about forming habits... potentially longer is better isn’t it I- I think to make it embedded properly and to stick... otherwise you might go yeah that’s - that’s enough for me for now.” (Ryan, Crohn’s disease) |
|  |  | Balanced cost | “I think you can talk about an opportunity cost what would they be doing if they weren’t doing this what would they be losing out on … as a time point of view the opportunity costs … uh that will be important to a sector of the population for example now I should be reading about ten research videos so that’s the opportunity cost … everybody has an opportunity cost.” (Ian, Gastroenterologist)  “Well then you’re dealing with a cohort who, you know, have a lot of associated costs in terms of medication and other things. And, and it may depend on your demographic. Like the demographic here at the suburban hospital is much poorer socioeconomic demographic. So, you know, for instance where we have patients that we refer to the GP to get a mental health plan. Um, they often don’t go to the GP, and if they do go to the GP and get a plan they often don’t enact it. And if they do enact it and there’s out of pocket costs, they don’t keep it going.” (Maggie, IBD nurse)  “If it’s something that would benefit me and I could get um assistance and I know that it’s something that will get me to my own better wellbeing then yeah I would pay but … again it would just depend on the cost and what I would get for it” (Ellen, Crohn’s disease) |
|  |  | Who has responsibility | ‘All those things are less of a priority on my list so sometimes I don’t get to them because it’s not much of a priority’ (Chelsea, gastroenterologist)  “I probably am you know I’m happy to go there but at the end of the day I probably don’t have the training to do it properly.” (Nicole, IBD nurse)  “Overwhelmed, because I’m overwhelmed already. I drown on a daily basis and I’m not just below the water I’m in the ocean depths.” (Maggie, IBD nurse)  “I think probably like one of the reasons I haven’t had much success in therapy is because a lot of like pretty much all the things I’m going to therapy for are to do with my Crohn’s, and I would spend the hour like explaining Crohn’s to them um and like obviously it’s not their fault they don’t know what it is, it’s not their job to, but they wouldn’t have much time to give me skills to cope with it.” (Amelia, Crohn’s disease) |
| Theme 2: Buy-in, credibility and trust | Demonstrates how both patients and health professionals explained that the program must appear credible and trustworthy. It was emphasised this was especially needed when encouraging patients to sign on to the intervention as this perceived credibility would increase the likelihood that they were willing to try it, and trust that they would get something meaningful and useful from the program. |  | “Umm having a source of truth as well from a credible source cos there’s so much RUBBISH out on the internet that you could read but its unqualified opinion.” (Ryan, Crohn’s disease) |
|  |  | Visual Appearance | “I like to read but- I mean I know that especially these days um people see a whole lot of... sentences and paragraphs and especially people who don’t like reading... are very visual and if there’s too much visually it just seems like too hard.” (Jack, Ulcerative colitis)  “I think that like I feel like it was written by somebody that doesn’t have fatigue” (Tiffany, Crohn’s disease)  “when you look at all the apps that are developed these days and what goes into them just how user friendly they are and sometimes they have little animations and things like that so they’re really great to grab people’s attention so … if they had a bit … more colour or things to grab people’s attention a couple more pictures or diagrams that would obviously help” (Justine, Psychologist) |
|  |  | The need for a deep understanding of IBD | “I think it’s really important for any tool that’s developed to consumer test it before it’s implemented more widely … and … should be prompted by the consumer” (Ian, Gastroenterologist)  “I’d like it to be someone who can relate to IBD so someone who has either studied it for a long period of time or someone like yourself who is doing this research” (Ellen, Crohn’s disease) |
|  |  | Rapport building | “You’re not just a number … like if you have to walk into the room and they’ve forgotten everything about you and they have to read your file type thing … like it kind of feels like they don’t care” (Tiffany, Crohn’s disease)  “Can a program communicate care?” (Sam, Clinical psychologist).  “In terms of whoever delivers the intervention, being able to connect with that person, and the emotional support, the validation, umm teasing things out in a bit more detail and a bit more personalized to them” (Sarah, Health Psychologist) |
|  |  | Evidence-based | “It needs to be endorsed by a credible body to be … to have an impact.” (Ian, Gastroenterologist)  “So you look at it like I should do these things, because like it’s actually going to help me rather than someone telling you go for an hour walk every single day and you’re like… cool, like I’m not going to do that cause I don’t know why.” (Tiffany, Crohn’s disease) |
|  |  |  | “If you got something credible and that …I guess if you’re saying this is from academia or something else it could be peer reviewed or whatever else or have some basis in you know research wouldn’t it … so yeah it would be nice to have a credible source” (Ryan, Crohn’s disease)  “I think like how effective it is … like if it says oh yeah this works for like seventy or eighty percent of people, then I’d be like willing to pay more I guess”. (Kate, Ulcerative colitis) |
| Theme 3: Accountability | Represents how participants identified that engaging in a psychological program, while beneficial, would be difficult to adhere to. Both patients and health professionals alike suggested that juggling the intervention with other commitments (e.g., work, parenting requirements) would be challenging and therefore patients would require several strategies to help them remain engaged and accountable. |  | “Because then I’m made accountable … if you wrote up a plan for me then you’ve gone through the effort to do that plan and if I don’t do it then I’m accountable and telling you now I will do it if it’s incorporated into something … but if you say oh you know what just do it once a week then that won’t that won’t motivate me … I think it would motivate me if I was held accountable.” (Ellen, Crohn’s disease) |
|  |  | Social interaction | “Yeah, and I think just especially if it was like I don’t know you know an in-person thing. Say every I don’t know month or couple of months, then I’d probably be more motivated to keep it up cause I would like want to go to that.” (Kate, Ulcerative colitis)  “I think group sessions could be good for some people because then they’re with other people who are going through the same thing with the potential of opening up about their own feelings so um interacting with other people to see what they might be doing which might help.” (Nicole, IBD nurse) |
|  |  | Adherence Difficulties | “like if you had to engage with somebody I don’t think it would be overwhelming if you knew… that things were going to be spaced out, that you knew… when it’s coming up, know when it’s coming up … like I had to do a module for something completely unrelated to what we’re talking about, and I know I had to have them done by the 17^th^ of march … so I know the expectations that I had to do them by that date … and I know I better get cracking on the reading … so sometimes having a due by date is good.” (Ellie, Crohn’s disease)  “Yes because I can’t even get my patients to get blood tests done sometimes … if I can’t even get them to do a blood test sometimes then I can’t get them to do this … this is going to be even more difficult.” (Nicole, IBD Nurse)  “These patients are already seeing dieticians, rheumatologists, infectious diseases, dermatologists, their gastroenterology, their surgeon, their GP. So, you know, another appointment out of their lives is very difficult.” (Maggie, IBD nurse)  “Yeah and logging without a patient having to actually physically log it if in some way there was an app on their phone or a watch app or something where it’s being picked up automatically rather than them logging it … can I log that I walked a kilometre when I didn’t actually walk a kilometre.” (Chelsea, Gastroenterologist) |
| Theme 4: Accessibility and Equity | Encapsulates patient and health professionals shared perspective that the program must be as accessible, equitable, and flexible as possible to ensure that patients can adhere to the program more easily and complete it successfully. For example, participants suggested that the program could be accessible via computer and smartphone, having telephone check ins with the program facilitator and keeping any activities short. |  | “I reckon that phone calls would be good like for me personally … just cause I’m a bit busy and stuff like that, like phone calls would be because then I can do it if like I’m at home or whatever, like if I’ve had a busy day and then like I don’t want to go back out again and meet somebody, like I can do a phone conversation, like I can do it from home.” (Tiffany, Crohn’s disease) |
|  |  | Benefits and limits of self-led interventions | “I think the benefit of having an online format is that even if you are doing face to face with someone or group, that you have another resource that you can go back to that reinforces it and has additional information. I think it’s a valuable …. resource to have.” (Amelia, Crohn’s disease)  “Yes some written stuff to reflect on it afterwards I think SO YOU might go into a session ... have that session and then walk away and then ... might have lost it in the moment you might walk away and not retain it so … something to read through or guidance for these practices I know it sounds like a no brainer but... it’s good perhaps- that there’s some kind of hand out or some kind of that effect that you got after the session so you’ve still got something to refer to.” (Jack, Ulcerative colitis)  “it just depends because I live in a remote part of part of Victoria … so where I would have to travel to and when I would have to travel and for how long so I’m so so busy with work at the moment that if I was to do any of this it would be after hours … and my after hours are so precious to me at the moment now that I just want it to be about me.” (Ellen, Crohn’s disease) |
|  |  | Digital literacy | “I guess it’d be good like if you had… like FAQ sheets and stuff like that, like if you had a question or even like an online thing, like if you were stuck and needed help and like … your phone call or you’re like catch wasn’t for a little while, like you could just go online and ask a question and have somebody answer you … like you could go back to that and be like okay, I get it now, like I’m not stuck anymore.” (Amelia, Crohn’s disease)  “So I think that you always have to think about these things in terms of an intervention, especially for a patient with inflammatory bowel disease where they always have different appointments going on and yeah, and making sure they’re understanding what you’re doing in terms of the intervention and someone to ask questions to if needed.” (Chelsea, Gastroenterologist)  “I know just for example like where I work they’ve got all … the options to look for the uh um ... things online there’s also options to download PDF form and print it.” (Jack, Ulcerative colitis)  “I think if you gave this idea to people some might go I’m actually illiterate so they’ll just go away because they’ll just go well that’s no good to me and then they don’t want to and they miss out on people to help them because there are barriers … I think that it’s only a small proportion it might affect it might not affect anyone but there is going to be that challenge in life and we have to cater for that person.” (Nicole, IBD nurse) |
|  |  | Financial cost | “For people who aren’t you know financially in a good position you know they think of petrol the cost of parking and then you know they start worrying about if they a ticket and everything so I don’t know in some ways the session may be lost in you know that.” (Nicole, IBD nurse)  “That’s one of the number one things that you hear from them … they struggle with the cost and usually they’re going to so many appointments that there might be other health conditions that they’re managing as well … and the cost is a significant deterrent to any program that was going to cost them extra money.” (Sarah, Health psychologist) |
