# Supplementary material for: A Qualitative Template Analysis to Understand Patient and Practitioner Perspectives on a Psychological Intervention for Fatigue in Inflammatory Bowel Disease
Source: JGH Open. 2026 Feb 16;10(2):e70363. doi: 10.1002/jgh3.70363 (PMC12907766; doi:10.1002/jgh3.70363)
Supplement: Supplementary file 2 — Data S2: Supporting Information. [file JGH3-10-e70363-s002.docx]

# Supplementary Materials

## Reflexivity Statement

Regarding reflexivity, whereby researchers actively acknowledge how their position within the project may influence data interpretation, all authors resided in Australia and were experiencing the same COVID restrictions as participants and fall within the same healthcare system. However, the research included insider and outsider perspectives toward IBD fatigue. The primary researcher (CE) drew on her own experiences living with IBD to guide the interview process with patients and health professionals, which would have actively contributed to the interpretation of the data. AMW and PRG additionally have experience with IBD in a professional sense, and a personal sense (AMW), which contributed to the development of the project. Contrastingly, AK, LO and MFT served as outsider perspectives to the field and experience of IBD. The research team also contained expertise across a variety of disciplines and research areas, including health psychology (CE, AMW, LO), clinical psychology (LO), medicine (PRG), social psychology and qualitative research (AK) and online intervention and co-development intervention research (MFT), thus all shaping and contributing to the interpretation of the data in their own professional processes and expertise.

| Table 2  Interview schedule for patients and health professionals | |
| --- | --- |
| Questions for Patients | Questions for Health Professionals |
| *Introduce ‘Tame Your Gut’ website and cognitive behavioural therapy*  How do you feel about using a website as an intervention to manage your fatigue? | *Introduce ‘Tame Your Gut’ website and cognitive behavioural therapy*  What are your thoughts on using a website for a fatigue intervention? Do you think it’s easy to use? |
| What are your thoughts on the website visually? | What are some potential barriers that you can think of that might stop patients from using a website? |
| Can you think of anything/any barriers that would stop you from using a website for a fatigue intervention? | *show example CBT activities*  What do you think of these activities? Do you think they’re user friendly and easy to understand? |
| *Show example CBT activities*  What do you think about these activities that could be a part of an intervention program? | What are some things you like about these activities? What are some things you dislike about the activities? |
| Are there any barriers that would prevent you from doing activities like these as a part of a program? | Can you see patients using these activities and why/why not? |
| Is there anything you can think of that would improve these activities? | Who do you think should deliver these activities? |
| How do you feel about doing physical activity as a part of a fatigue program? | What are your thoughts about incorporating physical activity in a fatigue intervention? |
| What are some barriers that could prevent you from engaging in physical activity as a part of an online program? | What are some barriers that may prevent patients from doing physical activity as a part of an intervention? |
| Would you prefer to do the program mainly online or face-to-face? | How many weeks do you think the intervention should run for? Why? |
| What do you think the balance should be between face-to-face and online sessions? Why do you think this? | How many sessions/activities do you think is feasible for patients to do/attend per week? Why? |
| Who do you think should deliver the program/any activities? Why? | How many sessions do you think should be in-person versus online per week? Why? |
| How many weeks do you think the intervention should run for? Why? | What are some barriers you can see patients having that could impact their ability to participate in the intervention? |
| How much time could you spend per week on an intervention? | What are your thoughts on cost? How much should the program cost? |
| What barriers can you think of that would prevent you from completing the intervention? |  |
| Would you be willing to pay for an intervention? How much do you think would be feasible? |  |
|  |  |
